# Heart involvement in patients with systemic sclerosis—what have we learned about it in the last 5 years

**DOI:** 10.1007/s00296-024-05699-x

**Published:** 2024-08-27

**Authors:** Aleksandra Nadel, Maciej Nadel, Nina Taborska, Bartosz Stępień, Jakub Gajdecki, Olga Brzezińska, Aleksandra Opinc-Rosiak, Joanna Makowska, Anna Lewandowska-Polak

**Affiliations:** 1 Department of Rheumatology, Immunology and Internal Medicine, Medical University of Lodz, Lodz, Poland; 2 II Department of Cardiology, Medical University of Lodz, Lodz, Poland

**Keywords:** Systemic sclerosis, Heart failure, Cardiac, Primary heart involvement

## Abstract

This review provides a detailed examination of original research and previously published reviews regarding cardiovascular involvement in systemic sclerosis (SSc). Our study aims to evaluate the current understanding of SSc-associated heart involvement (SHI), focusing on its most prevalent forms, diagnostic methods and treatment options. A comprehensive search of PUBMED, Medline, Web of science, Scopus and DOAJ databases was conducted, involving articles published between January 2019 and August 2024, available in English, both original research and reviews. Additionally, the authors examined the references cited in the selected articles, reviewed relevant literature, and included key publications dating back to 2010. Systemic Sclerosis (SSc) is an autoimmune connective tissue disease characterized by skin and internal organs fibrosis with accompanying vasculopathy. SHI encompasses both primary and secondary cardiac disease with a prevalence rate of up to 39%. It constitutes one of the leading causes of death among affected individuals. Systemic sclerosis- primary heart involvement comprises a wide range of conditions including arrhythmias, heart failure, pericardial disease, valvular abnormalities, and myocardial inflammation. However, its subclinical course, often misinterpreted as other forms of cardiomyopathy, poses true diagnostic challenges, requiring diagnostic tools like transthoracic echocardiography with tissue Doppler echocardiography and cardiac magnetic resonance imaging. The review underscores the importance of SHI and a holistic approach to managing patients with systemic sclerosis. Furthermore, it emphasizes the need for further investigation into potential pathogenetic mechanisms and biomarkers crucial for targeted treatment to fully optimize recommendations for this patient subgroup.

## Introduction

**Fig. 1:**
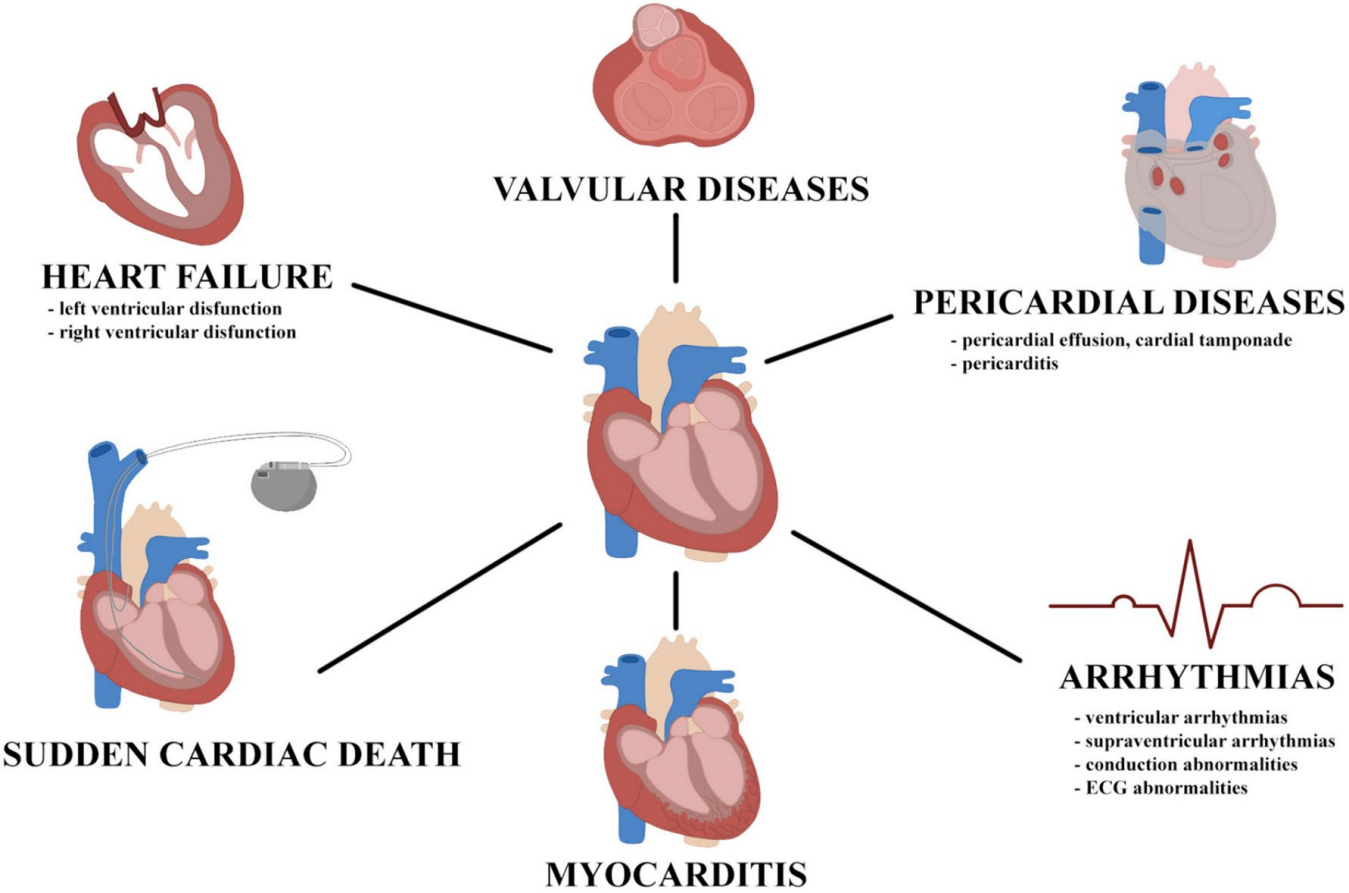
The most common manifestations of primary heart involvement in patients with systemic sclerosis

Systemic sclerosis (SSc) is a connective tissue disease (CTD) with pooled global prevalence of 17.6 per 100,000 individuals [1]. It manifests as an immune-mediated rheumatic disease, characterized by fibrosis of the skin and internal organs with concomitant vasculopathy [2]. Among affected structures, SSc-associated heart involvement (SHI) is one of the most frequent causes of death [3], including primary and secondary cardiac diseases [4]. Recently, two important papers on systemic sclerosis-primary heart involvement (SSc-pHI) emerged, providing both a clear definition of SSc-pHI and a practical guidance on screening, diagnosis and follow-up [5, 6]. A large survey conducted by European League Against Rheumatism Scleroderma Trials and Research (EUSTAR) showed that 12% of SSc deaths are attributed to primary heart disease. However, this determination was made by physicians, and specific SSc-pHI criteria were not provided to investigators during data collection [7]. Additionally, an extensive review uncovered a broad spectrum of estimated clinical prevalence rates for heart involvement in SSc, ranging from 7% to over 39%. This considerable variability underscores the significant heterogeneity in defining heart involvement across studies [8]. SSc-pHI can present clinically through a range of conditions, such as arrhythmias, heart failure, pericardial disease, valvular abnormalities, and myocardial inflammation. The most common forms of SSc-pHI are presented in Fig. 1. Heart involvement may persist subclinical or be misinterpreted as other forms of cardiomyopathy, posing challenges to accurate diagnosis [9, 10]. Detection of myocardial impairment can be achieved through a wide range of tools such as transthoracic echocardiography (TTE) with tissue Doppler echocardiography (TDE) or cardiac magnetic resonance imaging (CMR), offering extensive diagnostic opportunities. However, the prognostic implications of identifying “sub-clinical” fibrosis in this manner remain uncertain [11]. Additionally, the difference in applied diagnostic methods may contribute to epidemiological heterogeneity of SSc-pHI [11]. Moreover, the absence of precise guidelines for SSc-pHI treatment increases the complexity of therapy, making it more challenging and necessitating reliance on empirical procedures [12]. The aim of this study is to summarize recent knowledge about cardiac involvement in SSc, including definitions, screening, diagnostic options and treatment.

## Review methodology

**Fig. 2:**
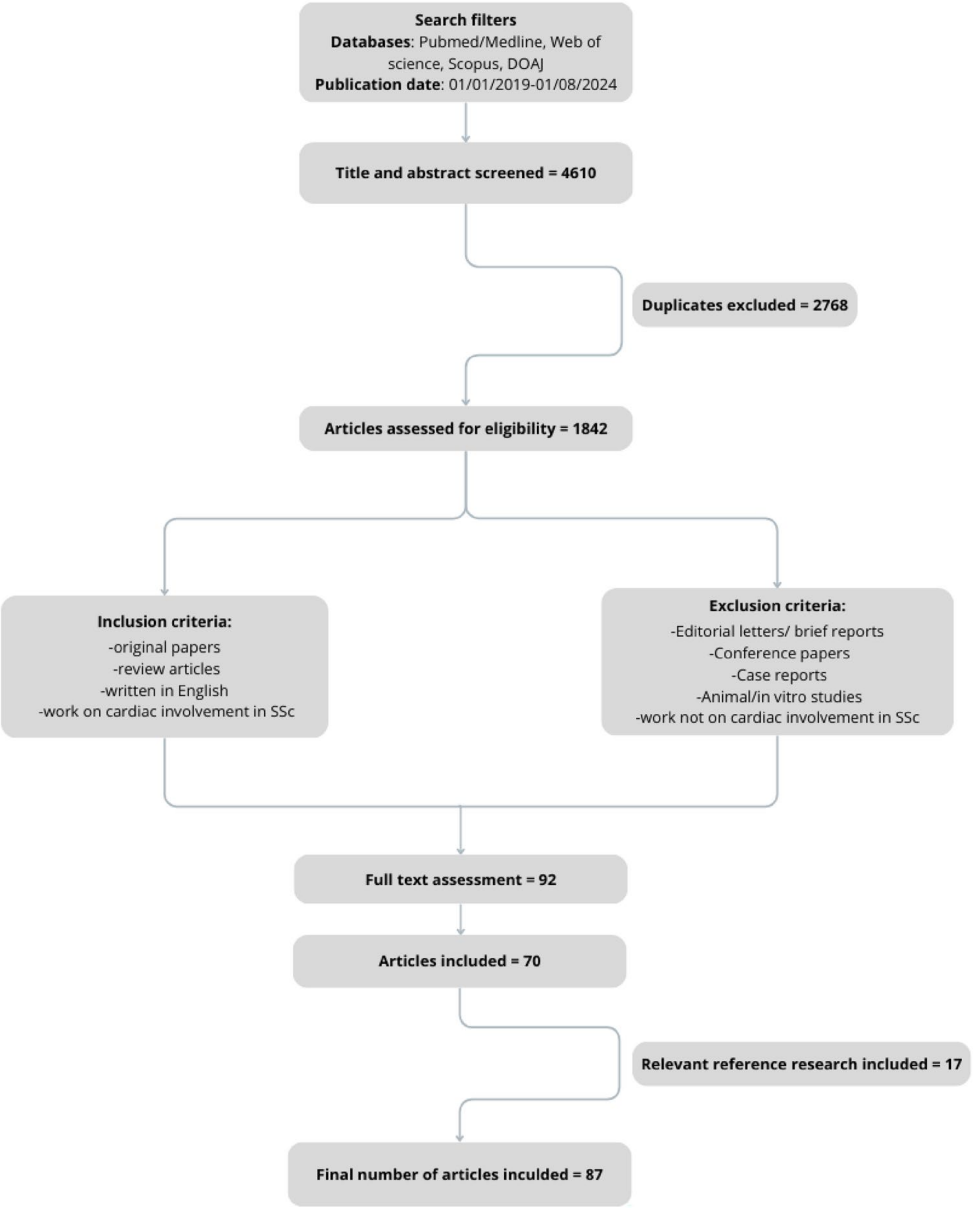
Screening and selection process

A literature search was conducted using the PUBMED, Medline, Web of science, Scopus and DOAJ databases, spanning from Jan 2019 to Aug 2024. The search terms employed were ‘cardiac’ OR ‘cardiovascular’OR 'heart' AND ‘systemic sclerosis’. Only published data were included, encompassing both original studies and reviews written in English. Animal or in vitro studies, case reports, editorial letters, and conference papers were not selected for further review. Abstracts and full articles were thoroughly reviewed and articles with study groups comprising patients with systemic sclerosis were selected for inclusion. Additionally, authors screened the references cited in the selected articles, reviewed the relevant literature and incorporated crucial publications until 2010. Figure 2 shows the screening and selection process.

## Pathophysiology of cardiac complications

The pathogenesis of cardiac complications in SSc is not fully understood, due to its complex nature [12, 13]. Small vessel damage, vasoconstriction, chronic ischemia–reperfusion injury, cardiac inflammation and fibrosis are critical mechanisms affecting each of the heart structures [11, 13, 14]. The “vascular hypothesis” (intermittent vascular spasm, ischemic necrosis, and reperfusion injury) is the most well-known mechanism leading to fibrogenesis. Recently, an inflammatory myocardial process resulting in fibrosis has been recognized as a crucial pathomechanism coexisting with vascular injury [15]. Among cytokines, interleukin 1 (IL-1) and interleukin 6 (IL-6) are crucial for cardiac inflammation and subsequent fibrosis, which may lead to the broader application of drugs targeting these points in the inflammatory cascade among patients with SHI [15].

## Risk factors

According to Bissell et al., several factors may be linked to heart involvement in SSc: male gender, diffuse cutaneous SSc (dcSSc), presence of anti-topoisomerase antibodies with rapid progression of skin thickness, anti-Ku, anti-Histone, anti-RNA polymerase, and anti-U3-RNP antibodies, age of onset over 65 years, presence of tendon friction rubs, digital ulcers, lung involvement and myositis [16]. All the above could potentially identify patients of a high risk for cardiac complications that require an exceptional clinical attention. Additionally, Ceribelli et al. determined that patients with anti-Th/To over anticentromere (ACA) antibodies had a greater prevalence of pericarditis [17]. In the study by Höppner et al., patients with SSc who were positive for antimitochondrial antibodies (AMA) M2 subunit, had an increased risk of cardiovascular events, independent of the presence of primary biliary cholangitis (PBC) [18].

## Primary heart involvement in systemic sclerosis

In 2022**,** Bruni et al. published an expert consensus definition for primary heart involvement in systemic sclerosis, being a response to the emerging need in the literature [5, 11]. According to this document “SSc-pHI comprises cardiac abnormalities that are predominantly attributable to SSc rather than other causes and/or complications (such as: non-SSc-specific cardiac conditions and/or SSc non-cardiac conditions). SSc-pHI may manifest as subclinical and requires confirmation through diagnostic investigation.

The pathogenesis of SSc-pHI comprises one or more of inflammation, fibrosis and vasculopathy” [5].

This standardized definition has enhanced our understanding and facilitated focus clinical research efforts on SSc-pHI. Furthermore, establishing clear diagnostic criteria is crucial for determining the prevalence of SSc-pHI, as its frequency remains unknown due to most existing studies not distinguishing between primary and secondary heart involvement [5]. Prior to the publication of SSc-pHI definition, Elhai et al. conducted a large survey through EUSTAR, revealing that 12% of SSc-related deaths were attributed to primary heart disease. However, patients were evaluated by physicians, and their opinions played a key role [7]. Hence, there is an unmet need to assess patients using novel criteria to determine whether SSc-pHI might contribute to varying death rates.

## Arrhythmias

Patients with SSc have an increased risk of conduction and rhythm disorders both at disease onset and over time, in comparison to individuals without SSc [19]. For instance, compared with seemingly healthy non-SSc cohorts, patients with SSc exhibit a 2.5-fold increase in electrocardiogram (ECG) abnormalities, and a twofold increase in conduction blocks [20]. The pathogenesis of arrhythmia remains complex, involving a combination of factors including the direct effects of microvascular injury, subsequent fibrosis development and autonomic dysfunction [9, 14]. A recent study conducted by Ross et al. found a high burden of myocardial fibrosis and arrhythmias among SSc patients without confirmation of evident cardiac disease; however, there was no clear association between focal or diffuse myocardial fibrosis and arrhythmias, indicating that CMR may have limited use as a screening tool to identify SSc patients at risk of future relevant rhythm disorders [21]. In a study conducted by Radwan et al. increasing age and pulmonary arterial hypertension (PAH) were associated with a higher risk of developing any conduction disorder in SSc, while preexisting PAH in addition to current smoking status were associated with higher risk of developing rhythm disorders [19].

**Fig. 3:**
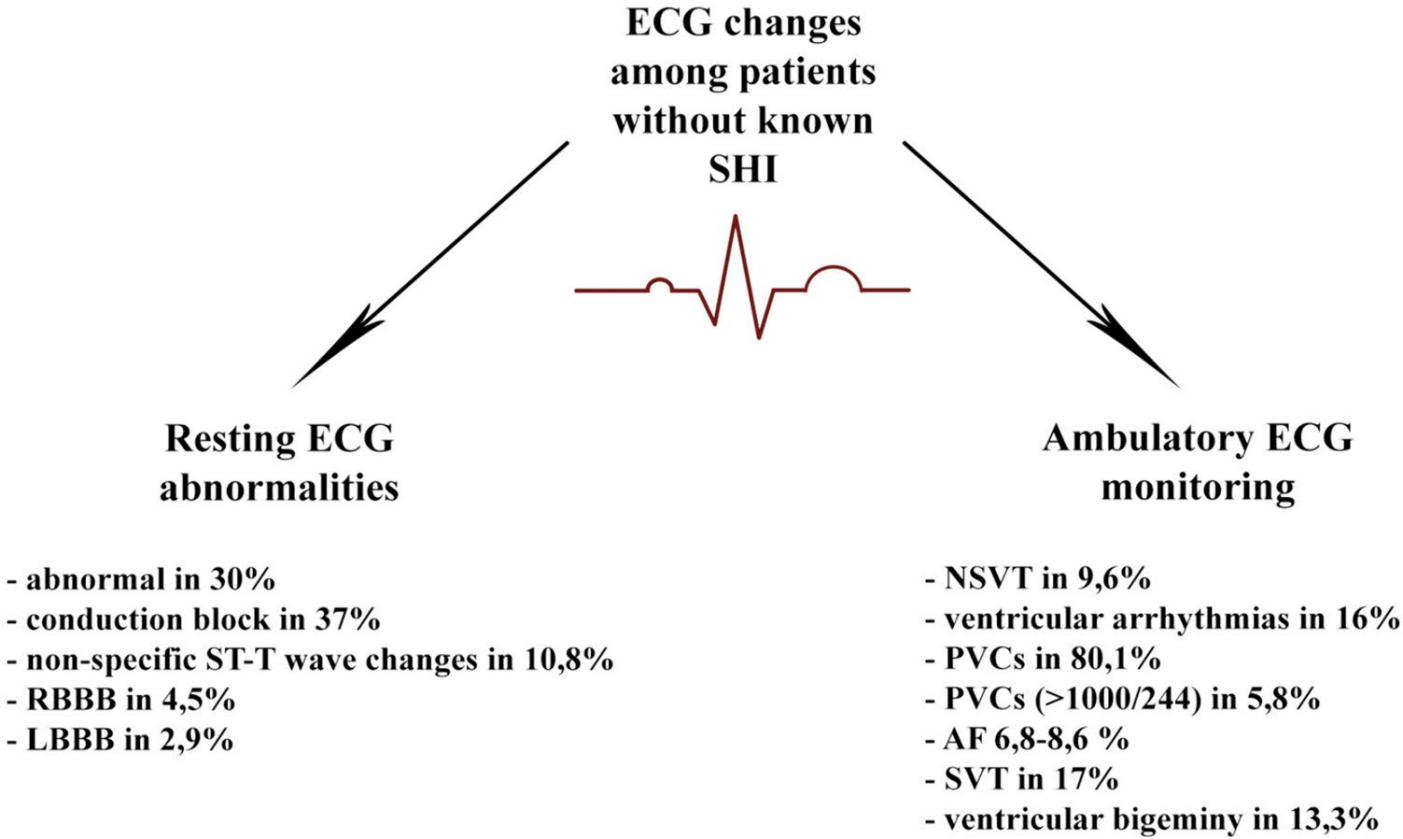
Resting and ambulatory ECG abnormalities among systemic sclerosis. *RBBB* right bundle branch block, *LBBB* left bundle branch block, *NSVT* non-sustained ventricular tachycardia, *PVCs* premature ventricular contractions, *AF* atrial fibrillation, *SVT* supraventricular tachycardia

Arrhythmias, according to European League Against Rheumatism (EULAR), account for 6% of deaths among patients with SSc [22].The patterns of arrhythmias and conduction disorders are various. Figure 3 shows a wide range of ECG abnormalities in SSc.

A large systematic review and meta-analysis revealed that among patients without known SHI, almost one-third had abnormal resting ECG. Conduction block occurred in 37% of them. Non-specific ST-T wave changes appeared in 10.8%. Right bundle branch block (RBBB) and left bundle branch block (LBBB) were less frequent, occurring in 4.5% and 2.9%, respectively. When heart involvement was reported, the rate of abnormalities was higher [20]. Additionally, in one case–control study, QT corrected for heart rate (QTc) interval in SSc patients was significantly higher than in the control group, with relationship between QTc and skin score of patients [23]. According to ambulatory ECG, non-sustained ventricular tachycardia (NSVT) occurred in 9.6% of SSc patients without known SHI, ventricular arrhythmias in 16.0%, ventricular bigeminy in 13.3%, premature ventricular contractions (PVCs) were detected in 80.1%, paired PVCs in 15.6%, and multiform PVCs in 44.4%. Frequent PVCs (> 1000/24 h) occurred in 5.8%, and > 100 PVCs/24 h in 23.5%. Paroxysmal atrial fibrillation (AF) was detected in 6.8%, combined AF in 8.6%, and paroxysmal supraventricular tachycardia (SVT) in 17.1% [20]. Moreover, in a study of 2519 patients with SSc, a two-fold higher risk of incident AF than controls had been reported [24]. Data from 13,609 individuals assessed an annual incidence of sudden cardiac death (SCD) in SSc cohorts to be between 1.0 and 3.3%. This illustrates at least a tenfold raise compared to general population [20, 25, 26]. Arrhythmias are indeed prevalent in SSc and can lead to increased mortality. However, they are not exclusive to this population and can occur in the general populace as well. Therefore, diagnosing and treating them early and accurately is challenging yet crucial for enhancing patient prognosis.

## Heart failure

In SSc, both left and right ventricles may be affected, leading to their dysfunction and failure. Heart failure (HF) is defined as a clinical syndrome with increasing prevalence that imposes a huge burden on health care systems worldwide. The pathomechanism of HF in SSc is still unclear, regarding myocardial fibrosis. Ongoing theories include vessel obliteration and arteriolar endothelial injury resulting in fibrosis [9]. Patients with SSc, compared with non-SSc individuals, have an increased risk of HF [27]. A study conducted among 1,830 SSc patients and 27,981 controls revealed that the cumulative HF rates at 1, 3, 5, and 10 years among patients with SSc were 1.3%, 3.5%, 5.3%, and 9.7%, respectively. In contrast, the cumulative incidence of HF at 1, 3, 5, and 10 years in the control group were 0.2%, 0.7%, 1.4%, and 3.1%, respectively [27]. Moreover, Sherif et al. in a recent study confirmed that among patients hospitalized due to HF, those with SSc had a higher likelihood of in-hospital mortality compared to those without SSc [28]. PAH and resulting right‐sided HF are a significant cause of morbidity and mortality in SSc [29–31]. The prevalence of left ventricle diastolic dysfunction (LVDD) varies, ranging from 17–21% at baseline to 29% after median 3.4 years follow-up [32, 33]. In a study conducted by Tennøe et al. the presence of LVDD among patients with SSc was associated with high mortality; however, assessing causality between diastolic dysfunction and death was not possible due to study design [32]. Although the occurrence of left ventricle systolic dysfunction (LVSD) appears to be rare, the data on its actual frequency within the SSc population is limited [34]. In a large cohort of 1141 participants with SSc without non-SSc cardiac disease or PAH, only 2.4% had a left ventricular ejection fraction (LVEF) < 50%, and 0.6% a LVEF < 40%. Survival and functional capacity were significantly worse in SSc patients with reduced LVEF, even after its improvement. However, reduced LVEF alone may underestimate the true frequency of systolic dysfunction in SSc [34]. In a study by Tennøe et al., with significantly smaller SSc patient group, LVEF was reduced in SSc patients compared with control individuals. A LVEF from 40 to 49% was observed in 8% of the entire cohort, while 3% had LVEF < 40% [32]. Based on the LVSD risk factors identified in the study by Werakiat et al., high mRSS, steroid use, and elevated creatine kinase (CK) level were associated with unimproved LVSD. In contrast, treatment with mycophenolate mofetil (MMF) might help prevent the progression of LVSD [35]. The abovementioned data highlight the urgent need for appropriate and early diagnosis and treatment of patients with SSc suspected or diagnosed with HF in order to prevent complications and death.

## Myocarditis

Myocarditis has been recognized as a potentially life-threatening aspect of myocardial involvement in SSc. However, the limited data hinders its diagnosis and treatment [36, 37]. In a study by De Luca et al. it was noted that SSc-associated myocarditis frequently presents with heart failure and severe dyspnea. Additionally, the extent of myocardial fibrosis on endomyocardial biopsy (EMB) was found to be higher compared to other forms of EMB-proven myocarditis and was linked to a poorer cardiac prognosis [37]. Currently, CMR is increasingly utilized to assess heart involvement in SSc, offering non-invasive and non-irradiating methods to identify cardiac disease through specific indexes and measurements [38]. A recent study has indicated that CMR can differentiate between reversible inflammatory or fibrotic lesions and irreversible fibrotic lesions among SSc patients with active myocarditis [39]. This underscores the role of CMR in myocarditis assessment, although guidelines regarding the use of CMR in monitoring treatment for SSc-associated myocarditis are lacking. Further research is necessary to establish appropriate diagnostic methods and treatment strategies in SSc myocarditis.

## Pericardial involvement

Pericardial effusion in SSc can manifest as either acute or chronic, with or without accompanying clinical symptoms. The prevalence of pericardial involvement varies, influenced by the criteria used for assessment. Autopsy studies often report a high occurrence, whereas echocardiography examinations commonly reveal lower frequencies [40]. In research conducted at a single tertiary center, clinically symptomatic pericardial effusions were observed in 6.9% of patients [40]. Cardiac tamponade was less common, occurring in 0.2% of patients with pericardial effusion, with a higher incidence among those with a history of atrial fibrillation. Patients with SSc might also develop constrictive pericarditis, however it is mainly described in the literature as case reports. Nonetheless, the true prevalence of pericardial involvement among SSc individuals may be higher [41–43]. Patients with pericardial effusion often present with pulmonary circulatory diseases, PAH, congestive heart failure, and end-stage renal disease. The presence of pericardial effusion and tamponade is associated with increased morbidity and mortality in SSc patients. Persistent pericardial effusion in SSc-PAH patients is linked to poorer survival outcomes [40, 43]. Various diagnostic tools are available to assess pericardial involvement, with echocardiography being a commonly used initial approach, however advancements in imaging modalities have provided additional diagnostic capabilities in recent years [44]. Considering the substantial mortality linked to these conditions, early identification and diagnosis of patients with comorbidities could be crucial in averting the advancement of pericardial effusion or pericardial tamponade. Nonetheless, additional research is required to determine the precise roles of the aforementioned characteristics and to establish diagnostic and treatment guidelines.

## Valvular diseases

Valvular heart disease (VHD) manifests as one of the SSc-associated cardiovascular complications, and its prevalence may be underestimated [45]. Traditionally, it was primarily linked with tricuspid regurgitation secondary to PAH. However, emerging literature suggests a broader spectrum of VHD presentations, encompassing all valves [45, 46]. The pathogenesis of VHD in SSc has not yet been extensively studied, however endocarditis-like changes on the mitral, tricuspid, or aortic valve in autopsy cases have been described [47].

According to a Danish nationwide cohort study, the relative risk of aortic stenosis (AS) is three times higher, aortic regurgitation (AR) four times higher, and mitral regurgitation (MR) five times higher in patients with SSc compared to the general population [30]. Although mild valve dysfunctions are common, particularly in the elderly, two studies focused on moderate and severe dysfunction degree. Kurmann et al. found a fourfold increase in the prevalence of moderate/severe VHD at SSc diagnosis compared to non-SSc patients, with a similar risk of developing moderate/severe VHD after SSc diagnosis. Moreover, SSc patients seemed to develop VHD prematurely [47, 48]. In another study by Narváez et al., the most prevalent moderate or severe valvular dysfunction was MR, observed in 5.2% of patients, followed by AS in 3.5% and AR in 1.7%. The prevalence of moderate to severe mitroaortic valve dysfunction was significantly higher in SSc patients compared to controls [46]. It's noteworthy that systematic echocardiography screening is recommended for SSc patients, which may facilitate early VHD detection. However, further studies are needed to establish this correlation definitively.

## Screening, diagnosis, and follow – up recommendation

For many years, efforts have been made to establish a consensus on the assessment of patients with SSc-pHI. Various algorithms have been developed to help in the detection, monitoring, and treatment of SSc-pHI patients [6, 16]. Among these, the UK Systemic Sclerosis Study Group was the first to offer guidance for physicians [16]. Recently, consensus-based guidelines have emerged, focusing on screening, diagnosis, and follow-up of SSc-pHI, with an emphasis on the role of multidisciplinary teams (MDT), that comprises cardiologists (with necessary subspecialist expertise as indicated) and rheumatologists with SSc expertise, moreover the importance of patient-doctor cooperation has been highlighted. It has been emphasized that both acute and chronic coronary syndromes (ACS and CCS) should be excluded when there is suspicion of SSc-pHI. In the event of their occurrence, they should be treated in accordance with current guidelines [6]. These guidelines facilitate clinicians' approaches to managing SSc-pHI patients and standardize the type and timing of procedures used to prevent or detect cardiac involvement early. Table 1 presents key consensus recommendations for clinical practice [6].

**Table 1:** Recommendations on assessment of patients with SSc-pHI based on consensus [6]

Characteristic	Symptomps and risk	Medical history and clinical examination	Laboratory biomarkers (hs-Troponin, NT-proBNP or BNP, ESR, CRP, CK)	ECG	TTE	CMR
pHI–SSc						
	Asymptomatic—low risk	Annual	Annual	Annual resting ECG	Annual	May be considered individually
Without established SSc-pHI diagnosis	Asymptomatic—high risk	Annual	Annual	Annual ECG; Holter may be considered	Annual	May be considered individually
	Symptomatic	Annual, unless other decision is made by the MDT	Annual, unless other decision is made by the MDT	Annual ECG/ ECG holter, unless other decision is made by the MDT	Annual	Should be considered if suspicion remains
Diagnosed with SSc-pHI		Annual, unless other decision is made by the MDT	Annual, unless other decision is made by the MDT	Annual, unless other decision is made by the MDT	Annual, unless other decision is made by the MDT	The MDT individual decision

*SSc-pHI* Systemic sclerosis primary heart involvement, *MDT* multidisciplinary team, *ECG* electrocardiogram, *TTE* transthoracic echocardiography, *NT-proBNP* n-terminal pro b-type natriuretic peptide, *hs-Troponin* high-sensitivity cardiac troponin, *ESR* erythrocyte sedimentation rate, *CK* creatine kinase, *CRP* C-reactive protein, *CMR* cardiac magnetic resonance

## Diagnostic options

There are numerous diagnostic methods employed in SHI, ranging from laboratory tests, resting ECG, and ambulatory ECG to TTE with TDE, speckle tracking echocardiography (STE) and CMR. These methods are incorporated into various algorithms, encompassing recent screening, diagnosis, and follow-up recommendations [6, 16, 49].

Evaluating the risk of heart complications in patients with autoimmune diseases, such as SSc is essential for identifying individuals at high cardiovascular (CV) risk and applying preventive measures [50]. Various tools, including the Framingham risk score (FRS) and QRISK3, have been utilized for this purpose [51].

The effectiveness of these scales in assessing CV risk in SSc patients was examined, with the study by Battista et al. suggesting that employing the QRISK3 algorithm among SSc patients could offer benefits by identifying individuals at high CV risk early, potentially capturing cases overlooked by conventional assessment scales [51].

### Echocardiography

Echocardiography, characterized by its wide availability, low cost, and safety, is a fundamental imaging method used in cardiological diagnostics. The echocardiographic screening for primary heart involvement should occur during the initial patient evaluation, with the appearance of symptoms indicating cardiac complications, and during the annual assessment of asymptomatic patients [6]. STE offers the ability to estimate myocardial deformation by measuring myocardial strain. This technique has become a routine element of echocardiographic assessment in many clinical situations such as monitoring cardiotoxic drug therapy or as an aid in diagnosing the etiology of certain cardiomyopathies [52, 53]. In recent years, many studies have emerged regarding the use of STE in assessing patients with SSc to search for primary heart involvement and associated myocardial fibrosis [54]. Impairment of left and right ventricular function assessed by global longitudinal strain (GLS), was observed in respectively 22.1% and 24.2% of patients with SSc without prior cardiac disease [55]. Importantly, only 2.1% of patients in this cohort had reduced LVEF. Basal and mid-segments of the anterior, lateral, and infero-basal walls were more frequently affected by abnormalities in GLS, and changes were more common in patients with dcSSc [55]. In contrast to LVEF, which does not seem to decrease over the course of the disease even in patients with its reduction in the initial assessment, GLS tends to decrease during several years of observation [56, 57]. A meta-analysis of 31 studies evaluating various deformation parameters showed that SSc patients exhibit worse values in a wide range of parameters such as left ventricular global longitudinal strain, left ventricular global circumferential strain, left ventricular global radial strain, right ventricular global wall strain, both left and right atrial reservoir/conduit strain, indicating the presence of impaired myocardium involving both the ventricle and the atrium [54]. In a recent study by Stronati et al., prospectively assessing the significance of left and right ventricular dysfunction assessed by GLS, it was shown that both LV GLS and RV GLS were independently associated with increased risk of death and hospitalization, and biventricular GLS impairment was associated with a ninefold increase in the risk of hospitalization compared to the group of patients with normal LV and RV GLS values [58].

### Cardiac magnetic resonance

CMR is increasingly utilized in cardiological diagnostics, allowing for the acquisition of both basic information regarding the morphology and function of the myocardium. This examination serves as a complementary method to echocardiography providing additional insights into myocardial structure. CMR has been able to detect both left and right ventricular diastolic dysfunction in patients with systemic sclerosis, even in those without significant abnormalities in echocardiographic examinations, potentially enabling a better assessment of early myocardial fibrosis associated with systemic sclerosis, although the clinical utility of these parameters must be evaluated in prospective studies [59]. CMR can also assess myocardial strain—among patients with newly diagnosed systemic sclerosis without cardiac symptoms, early assessment using GLS identified patients who had a greater disease severity during follow-up [60]. CMR is also capable of assessing myocardial perfusion—in patients with SSc, a reduced perfusion reserve after administration of adenosine or cold pressor testing compared to the control group, indicated subclinical microvascular dysfunction, though the prognostic significance of these parameters remains to be established in future studies [61, 62].

However, the strength of CMR is particularly evident in assessing parameters unavailable in echocardiography, such as late gadolinium enhancement (LGE), and increasingly utilized in clinical practice, parametric mapping [63]. While not completely specific, LGE serves as a reliable marker of myocardial fibrosis. Gadolinium-based contrast agents fill the interstitial space after administration and in normal conditions, the contrast remains in this space only for a short time due to efficient exit pathways. LGE can occur due to conditions that increase interstitial space or slow its exit, such as myocardial fibrosis [64]. In a group of patients undergoing CMR due to suspected cardiac involvement, 40% had presence of LGE [65]. A study of 344 SSc patients showed that LGE was present in 25% of patients and was significantly associated with the presence of digital ulcers and ventricular arrhythmias [66]. Moreover, patients with focal LGE fibrosis had significantly higher Rodnan skin scores compared to those without LGE [61].

In contrast to conventional techniques, parametric mapping allows for the visualization of tissue properties such as T1 and T2 relaxation times in a quantitative manner. These data, presented parametrically (in pixel form), allow for better tissue characterization, which can be utilized, for example, in the diagnosis of heart failure of unknown etiology [63]. Parametric mapping can assess the presence and extent of diffuse myocardial fibrosis and the presence of inflammation [67]. It has been shown that global T1 and T2 values are significantly higher in SSc patients compared to healthy control groups and abnormalities in native T1 and/or T2 times were present in 62% of patients without abnormalities in conventional CMR techniques such as LGE or T2 STIR [67]. It has also been shown that native T1 is a predictor of death from cardiovascular causes in SSc patients [68, 69].

Additionally, CMR mapping techniques improve the sensitivity of detecting myocardial inflammation in patients with SSc—in one study among patients with clinically confirmed myocarditis, the use of 2018 Lake Louis Criteria (LLC) for diagnosis of myocarditis which incorporates parametric mapping increased sensitivity from 52.6% to 89.5% compared to previous 2009 LLC criteria which were based on solely on conventional parameters [70].

Even though CMR is recommended as a diagnostic method in patients with suspected heart involvement, costs and the limited data on the clinical significance of abnormalities detected in asymptomatic individuals restricts its use in this population [6].

Nonetheless, in the coming years, we can expect an increasing role of CMR associated with the greater availability of advanced techniques and growing awareness of their role in the diagnosis of primary cardiac involvement in SSc.

### Positron emission tomography

Positron emission tomography (PET) is increasingly used in cardiology to assess metabolism and myocardial perfusion. Despite the absence of data on its utility in patients with SSc, there are some preliminary studies heralding its future role in the diagnosis of SHI. Myocardial flow reserve, assessed by PET/CT scan is reduced in patients with SSc and Reynaud phenomenon [71]. Moreover, a small study detected pathological fluorodeoxyglucose (FDG) uptake suggesting myocardial inflammation in 50% of asymptomatic SSc patients [72]. Recently, a new PET tracer developed for assessment of fibroblast activation [(68 Ga)Ga-FAPI-04], has been used in detection of myocardial fibrosis [73]. Its increased uptake was also present in SSc patients with arrhythmias, elevated serum N-terminal pro b-type natriuretic peptide (NT-pro-BNP), and LGE assessed by CMR [73]. However, the utility of PET in the diagnosis of SHI remains to be established in future research.

## Biomarkers

Assessing serum biomarkers offers a valuable, non-invasive method for screening SSc patients for cardiac involvement. Literature data suggests that NT-proBNP and high-sensitivity cardiac troponin (hs-Troponin) hold significant diagnostic and monitoring value for SHI, both pHI and secondary [6, 74, 75]. The abovementioned biomarkers could be useful in detecting subclinical cardiac involvement as well as predicting worse survival among SSc patients, exhibiting elevations in both NT-proBNP and hs-Troponin [74, 76]. However, considering hs-Troponin, it is worth mentioning that cardiac troponin I is regarded as specific to myocardial tissue, whereas troponin T (hs-cTnT) has been observed in regenerating skeletal muscle tissue as well [75]. Bosello et al. study noted a higher incidence of impaired systolic function, ECG abnormalities, and worse outcomes in SSc patients with elevated cardiac enzymes (hs-cTnT and NT-proBNP) [74]. Paik et al. similarly found that SSc patients with elevated troponin I face approximately double the risk of death compared to those without [75]. Wang et al. study demonstrated that NT-proBNP and C-reactive protein (CRP) independently predict death in SSc individuals [77]. The role of serum organ-specific anti-heart (AHA) and anti-intercalated disk autoantibodies (AIDA) as markers for autoimmune myocarditis in SHI was recently evaluated in a multicenter study. AHA and AIDA were more prevalent in SSc patients than in controls, with AHA positivity alone linked to their worse survival and increased mortality [78]. Moreover, novel biomarkers like angiopoietin 2 (ANGPT2), osteopontin (OPN), and tumor necrosis factor-related apoptosis-inducing ligand (TRAIL) appear to be associated with both left ventricular (LV) and right ventricular (RV) dysfunction in SSc [79]. However, despite their potential, anti-heart antibodies and these novel biomarkers have yet to feature in recent screening algorithms due to insufficient data on their clinical utility and limited clinical availability. [78, 80] In addition to typical cardiac biomarkers, CRP, Erythrocyte Sedimentation Rate (ESR), and CK have been proposed for annual assessment in SSc-pHI screening among unselected, stable/asymptomatic patients, serving as a non-specific workup that may indicate underlying cardiac disease [6].

## Treatment

In recent years more data on treatment options among patients with SHI is available and algorithms are evolving. The role of MDT, involving cardiologists, is highlighted. Table 2 summarizes the current approach to the treatment options. It is crucial to note that no randomized controlled trials have been conducted for SHI [81]. Moreover, in the absence of well-defined guidelines, the treatment of cardiac inflammation, fibrosis, and vasculopathy is carried out empirically [82].

**Table 2:** Current treatment options among systemic sclerosis patients with heart involvement depending on the clinical manifestation

Type of heart involvement	Treatment options
Myocarditis	DMARDs (such as: AZA, MMF, MTX, CYC) alone or with GCs [82, 83] Symptomatic treatment
Pericarditis	NSAIDs (carefully) Colchicine GCs [49]
Arrhythmia	Similar to the general population [12, 16, 82] B-blocker (carefully; cardioselective) Ivabradine (sinus tachycardia > 70/min) Oral anticoagulation, Pacemaker, ICD—if necessary [49, 82]
HFpEF	Diuretics for fluid retention Dapagliflozin/ Empagliflozin Treatment for an etiology, CV and non-CV comorbidities [49, 53, 84]
HFmrEF	Diuretics for fluid retention Dapagliflozin/ Empagliflozin ACEI/ ARNI/ ARB, MRA, B-blocker (carefully; cardioselective)—may be considered [49, 53, 84]
HFrEF	ACEI (carefully in SRC high risk patients)/ ARNI B-blocker (carefully; cardioselective) MRA Dapagliflozin/ Empagliflozin Loop diuretics for fluid retention CRT, ICD (in selected cases) [49, 53, 84]

*DMARDs* disease-modifying antirheumatic drugs, *AZA* azathioprine, *MMF* mycophenolate mofetil, *MTX* methotrexate, *CYC* cyclophosphamide, *GCs* glucocorticosteroids, *NSAIDs* non-steroidal anti-inflammatory drugs, *ACEI* angiotensin converting enzyme inhibitors, *ARNI* angiotensin II receptor-neprilysin inhibitor, *MRA* mineralocorticoid receptor antagonists, *SRC* scleroderma renal crisis, *CV* cardiovascular, *CRT* cardiac resynchronization therapy, *ICD* implantable cardioverter defibrillator, *HFpEF* heart failure with preserved ejection fraction, *HFmrEF* heart failure with mildly reduced ejection fraction, *HFrEF* heart failure with reduced ejection fraction

### Immunosuppression

According to French recommendations for the management of SSc immunosuppressive therapy is suggested for symptomatic heart disease with CMR-confirmed myocarditis [49]. A recent study conducted by De Luca et al. underscores the significance of immunosuppressive drugs in managing SSc-pHI patients. In cases involving myocarditis, immunosuppression should be considered essential: both pulse or oral corticosteroids in SSc myocarditis or pericarditis. Conventional disease-modifying antirheumatic drugs (DMARDs) such as azathioprine (AZA), MMF, methotrexate (MTX), and cyclophosphamide (CYC), either alone or in combination with glucocorticosteroids (GCs), have exhibited positive outcomes in SSc myocarditis. Among them, MMF stands out as the most commonly used, due to its safety profile, especially when weighed against the potential cardiotoxicity of cyclophosphamide [81, 82]. Usage of MMF is supported by the prospective cohort study, in which MMF improved cardiac function and clinical status used both as first-line agent, in patients with systemic rheumatic disease, such as SSc, and second-line therapy in those with isolated lymphocytic virus-negative or autoimmune myocarditis (VNM), intolerant or resistant to azathioprine, regardless of GCs dosage [83]. However further prospective randomized controlled trials are needed to confirm its efficacy in SSc myocarditis. Taking into consideration the important role of IL- 1 and IL-6 in pathogenesis of SSc heart involvement, anti-IL-1 anti IL – 6 drugs could be potential therapeutic option among SHI patients [12, 15]. However, data on other immunosuppression form, including abovementioned drugs and intravenous immunoglobulins (IVIG) administration are limited in the literature [82]. Regarding myocardial fibrosis, there is currently no therapy specifically designed to target it [82].

### Pericarditis

Following the recent French guidelines, in case of symptomatic pericarditis, treatment with non-steroidal anti-inflammatory drugs (NSAIDs), used carefully among patients with upper gastrointestinal problems, or colchicine may be offered as a first line of treatment. In rare cases high doses of GCs in combination with pericardial drainage may be justified [49].

### Arrhythmia

In the case of arrhythmia, the principles applied to the general population can be used in most cases [12, 16, 82]. Beta-blockers are not contraindicated, but their use is limited due to the risk of exacerbation of Raynaud's phenomenon and digital ulceration, with a preference for cardioselective ones. In case of sinus tachycardia > 70/min, ivabradine may be administered. Oral anticoagulant therapy is necessary according to current guidelines. Pacemaker implantation is recommended in case of significant conduction disturbances, as well as implantable cardioverter defibrillator (ICD) in SCD prevention [49, 82].

### Heart failure

In case of HF, conventional treatment should be administered when contraindications are not present, correspondingly to the LVEF and symptoms [12, 49, 82]. It includes loop diuretics (for fluid retention), beta blockers, angiotensin converting enzyme inhibitors (ACEi)/angiotensin II receptor blockers (ARB)/angiotensin II receptor-neprilysin inhibitor (ARNI), mineralocorticoid receptor antagonists (MRA), sodium glucose co- transporter-2 inhibitors (SGLT2i, such as dapagliflozin, empagliflozin) [53, 84]. Despite EUSTAR analysis, in which ACEi in SSc patients display a risk factor for scleroderma renal crisis (SRC), [85] they remain first choice in the treatment of HF, however, they should be used with great caution in SSc patients at high risk of SRC [82]. Heart transplantation is a therapeutic option in SSc with severe cardiac involvement; however, it has only been reported in several cases [86].

## Conclusions

Myocardial involvement is common in SSc and worsens the prognosis, being one of the leading causes of death among SSc patients [22]. Its management requires exceptional attention in clinical practice with timely and accurate diagnosis. Recently, comprehensive definition of SSc-pHI has emerged, providing structure to knowledge and advancing understanding of this manifestation. The new consensus on the assessment of SSc-pHI facilitated clinicians in their daily management of this patient subgroup. This visible progress in diagnostic approaches is promising for patient care, however continued research efforts are necessary to elucidate all potential pathogenetic mechanisms and identify biomarkers crucial for targeted treatment strategies. Additionally, the development of specific recommendations for SHI patients, encompassing therapeutic options, is essential to enhance their outcomes.

## Data Availability

This review is an original work and has not been copied or published elsewhere, in whole or in part, in any language.

## Declarations

### Editing agencies involved and the use of AI or editing software

This paper has been prepared by the authors without the involvement of editing agencies. No AI technologies or editing software were utilized in the creation of this work.

### Software tools and image banks

The figures and tables were created by authors using GIMP, Canva, and Microsoft Word. These tools provided essential features for designing and editing the graphics.
